# Transcarotid Access Versus Transfemoral Access for Transcatheter Aortic Valve Replacement: A Systematic Review and Meta-Analysis

**DOI:** 10.3389/fcvm.2021.687168

**Published:** 2021-05-27

**Authors:** Henri Lu, Pierre Monney, Roger Hullin, Stephane Fournier, Christian Roguelov, Eric Eeckhout, Vladimir Rubimbura, Laurent Faroux, Adelin Barrier, Olivier Muller, Matthias Kirsch

**Affiliations:** ^1^Service of Cardiology, Lausanne University Hospital and University of Lausanne, Lausanne, Switzerland; ^2^Cardiology Department, Reims University Hospital, Reims, France; ^3^Service of Internal Medicine, Lausanne University Hospital and University of Lausanne, Lausanne, Switzerland; ^4^Service of Cardiovascular Surgery, Lausanne University Hospital and University of Lausanne, Lausanne, Switzerland

**Keywords:** transcatheter aortic valve replacement, aortic valve stenosis, transcarotid, transfemoral, meta-analysis

## Abstract

**Background:** The transfemoral (TF) route is the gold-standard access for transcatheter aortic valve replacement (TAVR). In 10–15% of patients, alternative accesses are needed, such as the transcarotid (TC) access. We performed a meta-analysis to compare 30-day mortality and complications between TC-TAVR and TF-TAVR.

**Methods:** We searched PubMed/MEDLINE and EMBASE from inception to January 2021 to identify articles comparing TC-TAVR and TF-TAVR. Patients' baseline characteristics, procedural outcomes, and clinical 30-day outcomes were extracted.

**Results:** We identified 9 studies, among which 2 used propensity-score matching, including 1,374 TC patients and 3,706 TF patients. TC-TAVR was associated with significantly higher EuroSCORE II and Logistic EuroSCORE values (respectively 8.0 ± 6.7 vs. 6.3 ± 5.4, *p* = 0.002 and 20.8 ± 14.2% vs. 20.0 ± 13.4%, *p* = 0.04), a higher prevalence of peripheral artery disease (52.6 vs. 32.8%, *p* = 0.001), previous cardiac surgery (26.3 vs. 22.4%, *p* = 0.008) and coronary artery disease (64.6 vs. 60.5%, *p* = 0.020). The pooled results found TC-TAVR to be associated with a significantly higher 30-day mortality risk (RR, 1.41, 95% CI, 1.02–1.96, *p* = 0.040), and a lower rate of 30-day major vascular complications (RR, 0.48, 95% CI, 0.25–0.92, *p* = 0.030). No significant difference was found regarding permanent pacemaker implantation, major bleeding and acute kidney injury. A subgroup analysis of the two propensity-score matched studies found a statistically increased risk of 30-day neurovascular complications (RR, 1.61, 95% CI, 1.02–2.55, *p* = 0.040).

**Conclusion:** Compared with TF-TAVR, TC-TAVR was associated with an increased risk of 30-day mortality, likely related to a higher surgical risk and comorbidity burden, and with an increased risk of 30-day neurovascular complications. Careful preprocedural patient selection and close periprocedural neurological monitoring are paramount.

## Introduction

The transfemoral (TF) access is considered as the standard route for transcatheter aortic valve replacement (TAVR), due to its minimally invasive nature and to relatively low complication rates. However, it is not suitable in 10–15% of patients, mainly because of anatomical considerations: iliofemoral atherosclerosis, small or heavily calcified vessels, extreme vessel tortuosity or abdominal aortic aneurysms (1). Alternative accesses such as the transapical (TAp) (2), transaortic (TAo) (3), transcarotid (TC), and transsubclavian (TSc) ones (4, 5), have been developed for these settings. The TC access is interesting as it avoids thoracotomy and allows a direct and shorter pathway to the aortic valve from the puncture site, with the benefit of stable catheter delivery and improved movement precision (6). Several studies have suggested that the TC access might yield better periprocedural and 30-day outcomes than the “transthoracic” ones (TAp or TAo) (7–9), and even outcomes comparable to the TF access (10, 11). Some concerns remain regarding the risk of neurovascular complications associated with TC-TAVR, which could theoretically be higher due to direct injury to the carotid artery, embolic events during vessel manipulation, or transient reduction in blood flow during the procedure (11). So far, no guideline regarding the choice of the first-line alternative pathway exists, as the options depend on the patient's anatomy and local experience. Previous meta-analyses compared TC-TAVR to other alternative accesses (12, 13), while others analyzed the pooled results of TC and TSc accesses vs. the TF access (14). To our knowledge, no meta-analysis was conducted on the comparison of TC-TAVR alone vs. TF-TAVR.

The objective of this meta-analysis was to access the risk of 30-day all-cause mortality and other 30-day complications of TC-TAVR, compared with TF-TAVR.

## Methods

This study followed the guidelines of the Cochrane handbook for systematic reviews of interventions (15). The results were reported according to the Preferred Reporting Items for Systematic Reviews and Meta-Analyses (PRISMA) statement guidelines (16).

### Literature Search and Selection Criteria

A systematic literature search of all relevant data from inception to January 31st, 2021, was conducted via the online databases PubMed/MEDLINE (Medical Literature Analysis and Retrieval System Online) and EMBASE (Excerpta Medica Database), using the following keywords and Medical Subject Headings (MeSH): “transcatheter aortic valve implantation,” “transcatheter aortic valve replacement,” “TAVI,” “TAVR,” “transcarotid,” “carotid,” “transcervical.” The search strategy is presented in Supplementary Table 1. Studies were included if they met the following criteria: (1) original articles, (2) comparison of TC and TF-TAVR, (3) reported data on population characteristics, periprocedural and 30-day clinical outcomes. Abstracts, case series, review articles, meta-analyses, non-human studies, and non-English language publications were excluded. When 2 similar studies were found from the same author, the most recent one was included in the final analysis. In the case of overlapping populations (based on a common data registry), only the most recent study, with the biggest and most thorough population sample was included. The eligibility of studies was independently assessed by two authors (HL and SF), with any disagreement resolved by consensus, or with the help of a third senior author (MK). The Newcastle-Ottawa scale was used to assess the quality of each study (17).

### Outcomes

Pooled-data outcomes included 30-day all-cause mortality and 30-day complications: neurovascular complications [stroke or transient ischemic attack (TIA)], major vascular complications, major bleeding, permanent pacemaker implantation (PPM), and acute kidney injury (AKI). For each outcome, a subgroup analysis was performed with propensity-score matched studies only. Outcomes were defined as reported in the studies; whenever possible, the Valve Academic Research Consortium-2 (VARC-2) definitions were used (18).

### Statistical Analyses

Data were summarized using descriptive statistics, with medians and interquartile ranges (IQR) or means with standard deviation (SD) for continuous variables, and frequencies with percentages for dichotomous variables. When data were reported as medians with IQR, they were not incorporated in the comparison analyses as they supposedly did not follow a normal distribution. Meta-analyses were performed by combining the results of the published incidence of the predetermined outcomes. The relative risks (RR) and their 95% confidence intervals (CI) were used as summary statistics. The *I*^2^ statistic was used to estimate the percentage of total variation across studies due to heterogeneity rather than chance: intervals of <25%, 25–50%, and >50% were used to classify heterogeneity as low, moderate, and high. The random-effects model was used to account for population diversity and methodological variation among studies. All *p*-values were two-sided. Publication bias was assessed by examination of the funnel plots for each outcome (Supplementary Figure 1).

Statistical analyses were carried out using the SPSS 24.0 (SPSS Inc., Chicago, Illinois, USA) and Review Manager 5.4 (The Nordic Cochrane Center, The Cochrane Collaboration, Copenhagen, Denmark) softwares.

## Results

### Search Results

**Figure 1:**
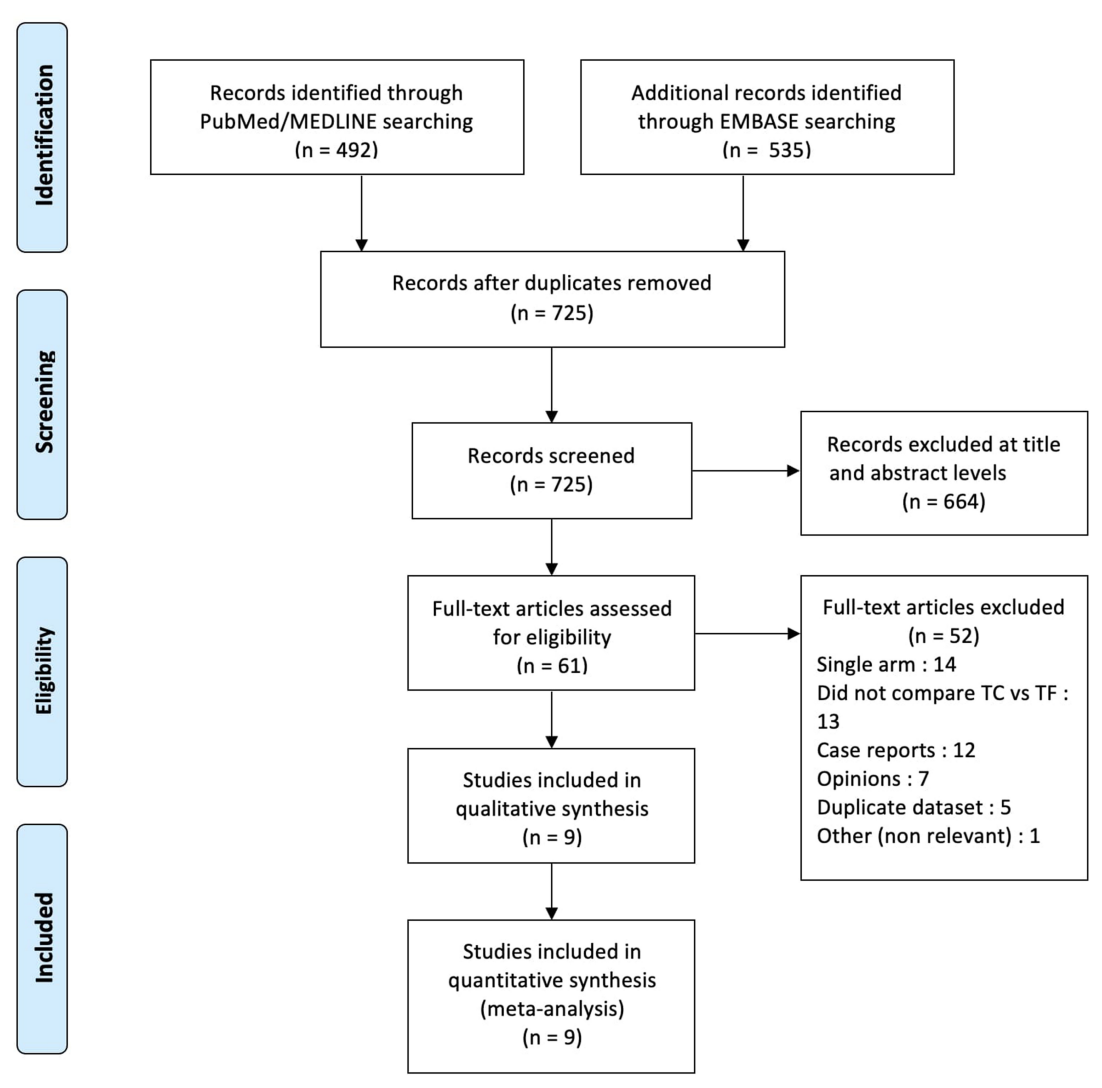
PRISMA flow diagram.

A total of 1,027 references were identified through the PubMed/MEDLINE and EMBASE databases. After removing duplicates, 725 publications remained; 664 were excluded after screening at titles and abstracts. Sixty-one full-text articles were assessed for eligibility, with a further 52 being excluded. A large registry-based study using propensity-score matching was excluded because of overlapping population with another study (19, 20): the same registry was used in both cases, and the study period of the first was included in the study period of the second. Nine articles were finally identified and selected. The PRISMA diagram presents the search strategy (Figure 1). All nine studies were observational and retrospective in nature. One study used data from a multicenter prospective data registry (20), and two studies performed propensity-score matching (20, 21). In two studies, TC and TSc patients were pooled in one same extra-thoracic pathway group and compared with TF patients (20, 21): in the first case, Supplementary Materials were available online and were used to analyze TC patients; in the second case, two co-authors provided the data regarding TC patients. Following a quality assessment of each study, seven publications were considered high-quality and two publications medium quality, all being suitable for inclusion in the meta-analysis (Supplementary Table 2).

### Study and Patient Characteristics

The nine studies included a total of 5,080 patients (TC-TAVR: 1374, TF-TAVR: 3706). Baseline patient characteristics, main comorbidities and surgical risk are presented in Table 1, while comparisons between the TC group and the TF group are presented in Table 2 (dichotomous variables) and Table 3 (continuous variables). There were differences regarding the way surgical risk was assessed: the EuroSCORE II was reported in 5 studies (11, 21–24), the Society of Thoracic Surgeons (STS) score in 6 (11, 22–27), and the Logistic EuroSCORE in 2 (20, 22). Overall, TC patients had a significantly higher prevalence of peripheral artery disease (PAD) (52.6 vs. 32.8%, *p* = 0.001), previous cardiac surgery (26.3 vs. 22.4%, *p* = 0.008), and coronary artery disease (64.6 vs. 60.5%, *p* = 0.020). TC patients presented a significantly higher surgical risk, as assessed by the EuroSCORE II and the Logistic EuroSCORE (respectively 8.0 ± 6.7 vs. 6.3 ± 5.4, *p* = 0.002 and 20.8 ± 14.2% vs. 20.0 ± 13.4%, *p* = 0.04); there was no significant difference regarding the STS score (6.9 ± 4.4 vs. 6.4 ± 4.3, *p* = 0.29).

**Table 1:** Characteristics of patients undergoing transcarotid transcatheter aortic valve replacement.

**References**	**Study arm**	**Sample size**	**Age (years)**	**Male gender (%)**	**Surgical risk**					**Comorbidities (%)**				
					**EuroSCORE II**	**STS score**	**Logistic EuroSCORE**	**HTA**	**CAD**	**Previous cardiac surgery**	**Diabetes**	**PAD**	**COPD**	**Stroke/TIA**
Kirker et al. (25)	TC TF	25 100	77.0 (72.0–83) 83.0 (79.0–88.0)	52.0 51.0	Unknown Unknown	6.1 (4.1–9.6) 6.0 (4.4–8.1)	Unknown Unknown	88.0 85.0	Unknown Unknown	44.0 37.0	48.0 34.0	80.0 39.0	28.0 19.0	16.0 13.0
Paone et al. (27)	TC TF	32 373	79.0 ± 9.6 80.4 ± 9.2	50.0 55.0	Unknown Unknown	6.9 ± 4.4 6.1 ± 4.3	Unknown Unknown	93.8 91.4	Unknown Unknown	Unknown Unknown	34.4 41.0	78.1 23.3	62.5 27.4	40.6 21.2
Watanabe et al. (22)	TC TF	83 643	80.0 ± 7.5 81.4 ± 8.4	65.1 53.7	8.2 ± 6.7 6.4 ± 5.5	6.4 ± 3.3 6.7 ± 4.3	24.2 ± 13.3 21.3 ± 12.4	80.7 75.1	Unknown Unknown	24.0 23.4	31.3 26.9	61.4 20.5	34.9 36.2	9.6 11.8
Beurtheret et al. (20)	TC TF	911 1613	81.6 ± 7.8 82.1 ± 7.6	60.0 63.3	Unknown Unknown	Unknown Unknown	20.54 ± 14.26 19.43 ± 13.81	Unknown Unknown	63.9 62.4	26.3 22.1	30.3 29.1	52.6 49.9	23.1 25.1	12.6 13.6
Villecourt et al. (21)	TC TF	32 40	86.0 (79.2–88.0) 84.0 (81.0–87.0)	50.0 42.5	2.9 (2.0–4.4) 3.2 (2.1–4.5)	Unknown Unknown	Unknown Unknown	75.0 80.0	43.7 47.5	Unknown Unknown	31.2 42.5	46.9 37.5	15.6 17.5	12.5 12.5
Junquera et al. (26)	TC TF	127 399	78.0 (72.0–82.0) 82.0 (72.0-86.0)	57.5 56.9	Unknown Unknown	4.7 (3.2–6.8) 4.2 (2.8–6.7)	Unknown Unknown	89.8 84.2	74.0 59.9	Unknown Unknown	44.1 32.1	47.2 10.5	33.9 22.3	11.8 10.0
Lu et al. (11)	TC TF	51 255	83.0 (80.0–85.0) 83.0 (79.0–87.0)	60.8 49.8	3.9 (2.7–5.9) 3.3 (2.0–5.7)	4.06 (3.1–6.6) 3.0 (2.1–4.9)	Unknown Unknown	70.6 75.7	62.7 52.1	23.5 11.0	27.5 24.3	41.2 14.1	17.6 12.9	21.6 11.4
Leclercq et al. (23)	TC TF	80 51	81.6 ± 7.5 81.8 ± 6.3	68.8 33.3	7.8 ± 8.6 5.5 ± 3.4	7.3 ± 5.4 4.5 ± 2.5	Unknown Unknown	Unknown Unknown	Unknown Unknown	22.5 19.6	36.3 42.0	Unknown Unknown	Unknown Unknown	12.5 11.8
Hudziak et al. (24)	TC TF	33 232	77.0 (72.0–85.0) 79 (74–83)	51.5 43.5	6.0 (4.8–10.7) 4.8 (2.8–7.9)	Unknown Unknown	Unknown Unknown	100.0 90.1	Unknown Unknown	30.3 28.9	57.6 43.1	36.4 18.1	24.2 12.5	3.0 11.2

*Age, EuroSCORE II, and STS score are expressed as mean ± SD or median (IQR). STS, Society of Surgeons; HTA, hypertension; CAD, coronary artery disease; PAD, peripheral artery disease; COPD, chronic obstructive pulmonary disease; TIA, transient ischemic attack*.

**Table 2:** Comparison of dichotomous patient characteristics between TF-TAVR and TC-TAVR cohorts.

**Characteristics**	**Number of studies (references)**	**TC-TAVR**		**TF-TAVR**		***P*-value**
		***n***	**%**	***n***	**%**	
Male gender	All (11, 20–27)	822	59.8	2,115	57.1	0.077
Comorbidities						
•HTA	7 (11, 21, 22, 24–27)	326	85.1	1,676	82.1	0.150
•CAD	4 (11, 20, 21, 26)	724	64.6	2,307	60.5	0.020
•Previous cardiac surgery	6 (11, 20, 22–25)	311	26.3	649	22.4	0.008
•Diabetes	All (11, 20–27)	453	33.0	1,153	31.1	0.206
•PAD	8 (11, 20–22, 24–27)	680	52.6	1,199	32.8	<0.001
•COPD	8 (11, 20–22, 24–27)	331	25.7	916	25.1	0.712
•TIA/stroke	All (11, 20–27)	193	14.0	493	13.3	0.491

*STS, score: Society of Surgeons score; HTA, hypertension; CAD, coronary artery disease; PAD, peripheral artery disease; COPD, chronic obstructive pulmonary disease; TIA, transient ischemic attack; TC, transcarotid; TF, transfemoral; TAVR, transcatheter aortic valve replacement; M-H, Mantel-Haenszel*.

**Table 3:** Comparison of continuous patient characteristics between TF-TAVR and TC-TAVR cohorts.

**Characteristics**	**Number of studies (references)**	**TC-TAVR**	**TF-TAVR**	**Mean difference (95% CI)**	***P*-value**
Age (years)	4 (20, 22, 23, 27)	81.4 ± 7.8	81.7 ± 8.0	−0.59 (−1.15, −0.02)	0.04
**Surgical risk**
•EuroSCORE II	2 (22, 23)	8.0 ± 6.7	6.3 ± 5.4	1.97 (0.75, 3.19)	0.002
•STS score	3 (22, 23, 27)	6.9 ± 4.4	6.4 ± 4.3	1.05 (−0.89, 2.98)	0.29
•Logistic EuroSCORE (%)	2 (20, 22)	20.8 ± 14.2	20.0 ± 13.4	1.44 (0.08, 2.80)	0.04

*TC, transcarotid; TF, transfemoral; TAVR, transcatheter aortic valve replacement. Studies where data were reported as medians (IQR) were not incorporated in the comparison analyses as they supposedly did not follow a normal distribution*.

Finally, TC patients were significantly younger than TF patients (81.4 ± 7.8 vs. 81.7 ± 8.0 years, *p* = 0.04). There was no significant difference regarding gender, hypertension, chronic obstructive pulmonary disease, diabetes, or history of neurovascular disease.

### Technical Aspects of TC-TAVR

Although TC-TAVR interventions were performed in heterogeneous ways in the 9 studies, some convergent points could be found: all cases used a surgical approach, with a 4–7 cm incision made along the anterior border of the sterno-cleido-mastoid muscle, which was then retracted to expose the common carotid artery. Subsequent technique for artery puncture and transcatheter heart valve (THV) placement then depended on the THV type that was used and local experience. Some technical aspects of TC-TAVR are presented in Supplementary Table 3. Self-expendable (SE) THVs were used in 52.7% of cases. All SE THVs belonged to the Medtronic CoreValve family (Medtronic, Minneapolis, MN, USA). Almost all remaining cases benefited from the Edwards SAPIEN family THVs (Edwards Lifesciences, Irvine, CA, USA), except one, reported by Hudziak et al., in which an Abbott Portico THV (Abbott Vascular, Santa Clara, CA, USA) was implanted (24). 70.8% of interventions were performed via the left common carotid artery (CCA). General anesthesia was used in 99.1% of cases. Finally, continuous cerebral monitoring via cerebral oximetry was reported in all but three studies: two did not describe cerebral monitoring (20, 23), while one reported not using cerebral monitoring (27). Supplementary Table 4 shows the proportion of SE and balloon-expandable (BE) THVs used in TC and TF-TAVR interventions: SE THVs were more frequently used in the TC group compared with the TF group (46.3 vs. 36.0, *p* < 0.001).

### Meta-Analysis of Outcomes

Thirty-day complications of TC-TAVR and their respective incidence are presented in Supplementary Table 5.

#### Thirty-Day All-Cause Mortality

**Figure 2:**
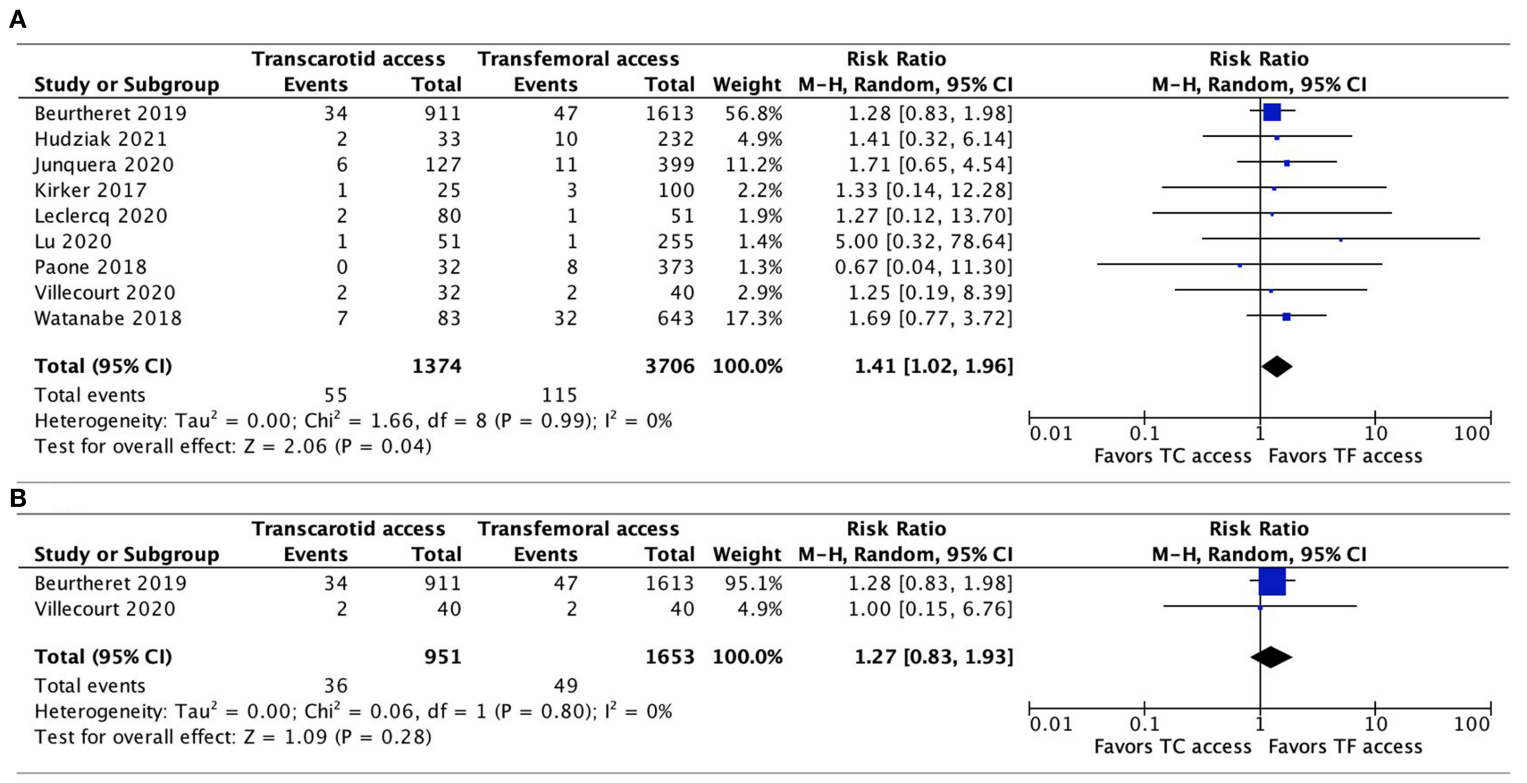
Forest plots comparing 30-day all-cause mortality rates between TC and TF transcatheter aortic valve replacement procedures. **(A)** Pooled data from all studies. **(B)** Pooled data from propensity-score matched studies. TC, transcarotid; TF, transfemoral.

Data regarding 30-day mortality were provided by all 9 studies. The pooled results found TC-TAVR to be associated with a significantly higher mortality risk (RR, 1.41, 95% CI, 1.02–1.96, *p* = 0.04), with no heterogeneity (*I*^2^ = 0%; Figure 2A). However, in a subgroup pooled analysis of the 2 propensity-score matched studies (20, 21), this association was no longer found (RR, 1.27, 95% CI, 0.83–1.93, *p* = 0.28, *I*^2^ = 0%; Figure 2B).

#### Neurovascular Complications

**Figure 3:**
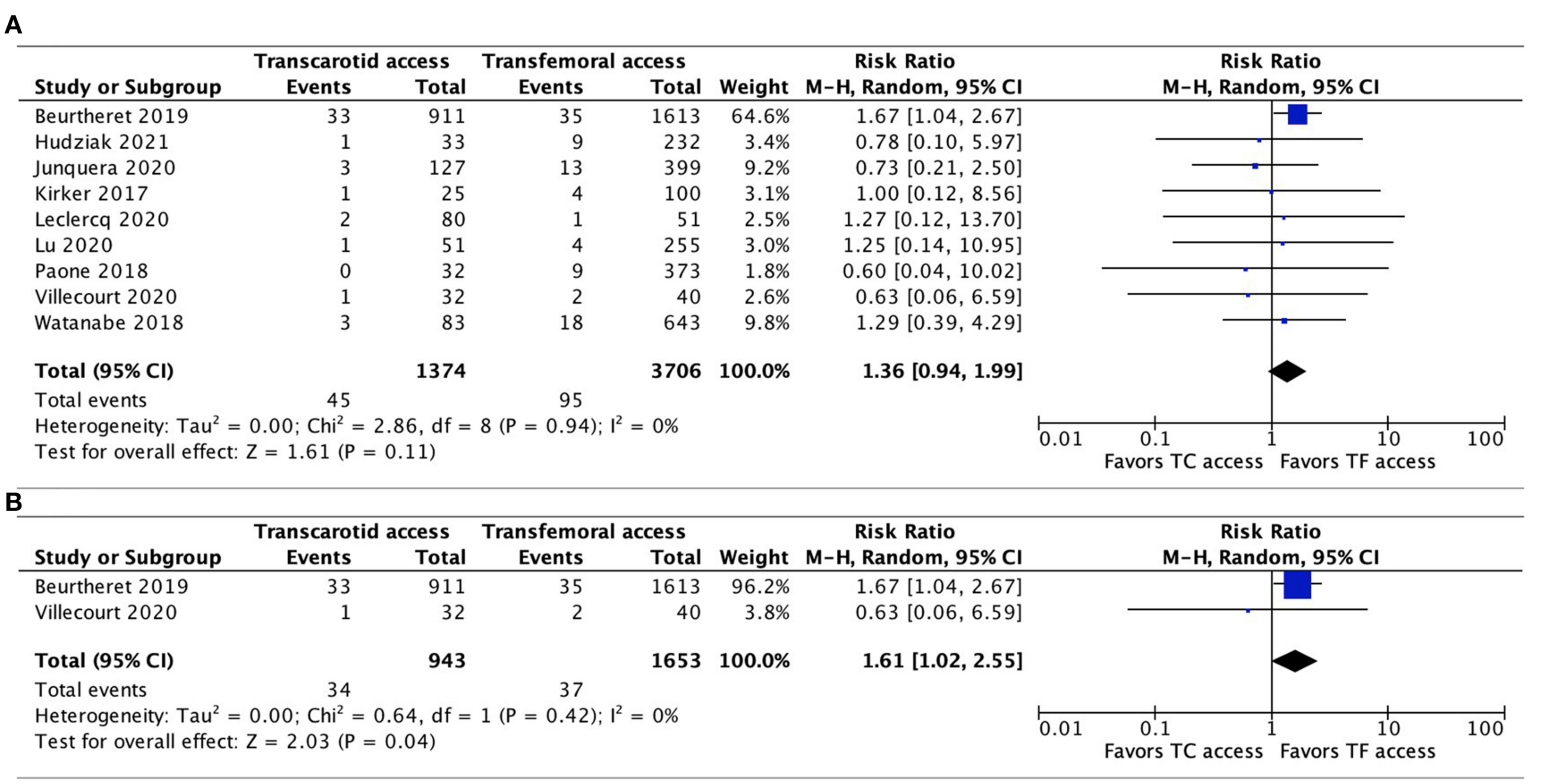
Forest plots comparing neurovascular complications at 30 days between TC and TF transcatheter aortic valve replacement procedures. **(A)** Pooled data from all studies. **(B)** Pooled data from propensity-score matched studies. TC, transcarotid; TF, transfemoral.

Data regarding 30-day neurovascular complications were available in all 9 studies. The pooled results showed a trend toward a higher risk of neurovascular complications associated with TC-TAVR, without reaching statistical significance (RR, 1.36, 95% CI, 0.94–1.99, *p* = 0.11), and with no heterogeneity (*I*^2^ = 0%; Figure 3A). Interestingly, in the subgroup pooled analysis of the two propensity-score matched studies (20, 21), the association between TC-TAVR and neurovascular complications was statistically significant (RR, 1.61, 95% CI, 1.02–2.55, *I*^2^ = 0%, *p* = 0.04; Figure 3B).

#### Major Vascular Complications

**Figure 4:**
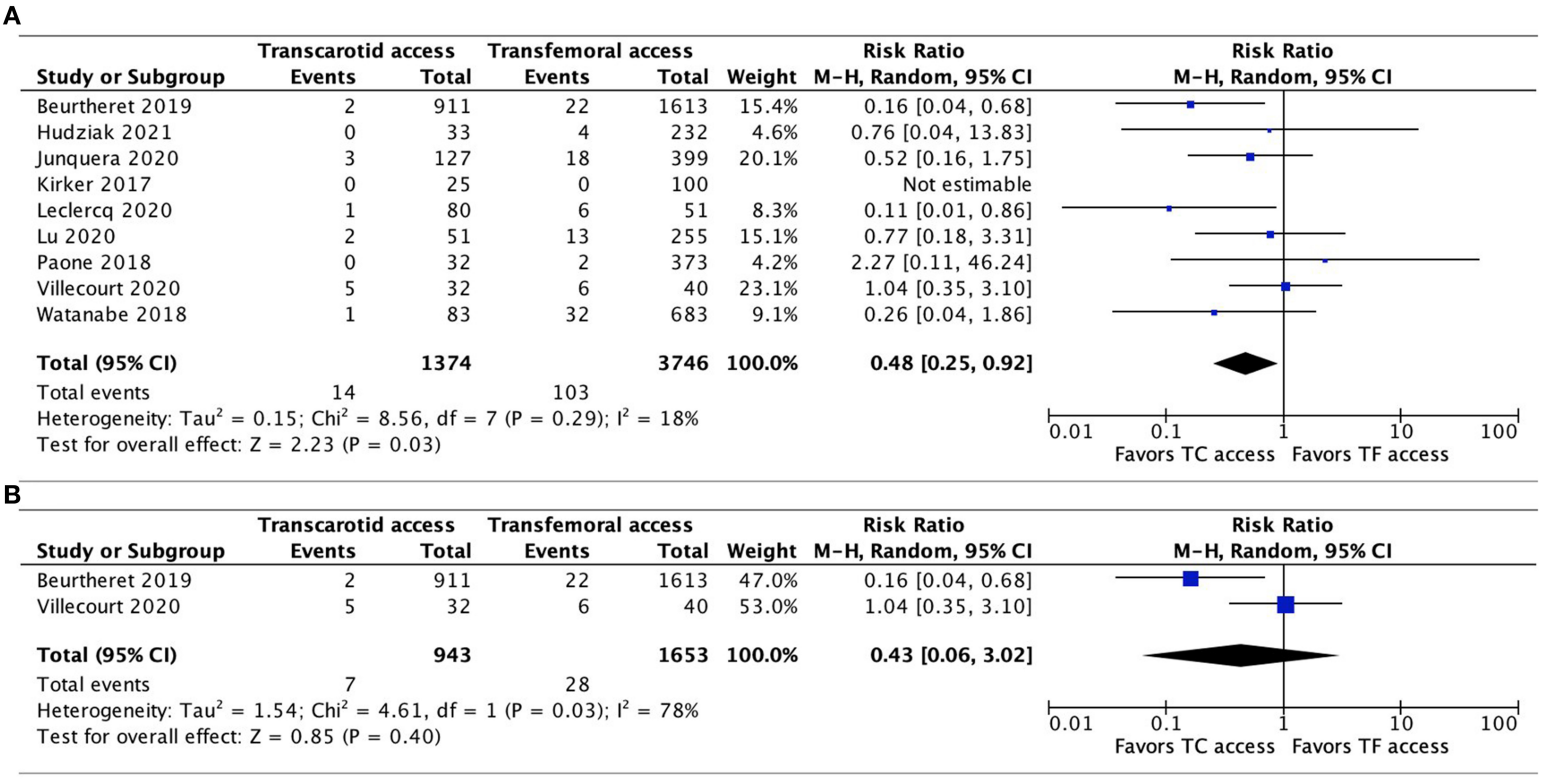
Forest plots comparing major vascular complications between TC and TF transcatheter aortic valve replacement procedures. **(A)** Pooled data from all studies. **(B)** Pooled data from propensity-score matched studies. TC, transcarotid; TF, transfemoral.

Data on the incidence of major vascular complications were available in all 9 studies. In the pooled analysis, TC-TAVR was associated with a lower risk of major vascular complications (RR, 0.48, 95% CI, 0.25–0.92, *p* = 0.03; Figure 4A). Heterogeneity was low (*I*^2^ = 18%). In the subgroup pooled analysis of the 2 propensity-score matched studies (20, 21), this association was no longer found (RR, 0.43, 95% CI, 0.06–3.02, *p* = 0.40), but heterogeneity was high (*I*^2^ = 78%; Figure 4B).

#### Permanent Pacemaker Implantation

**Figure 5:**
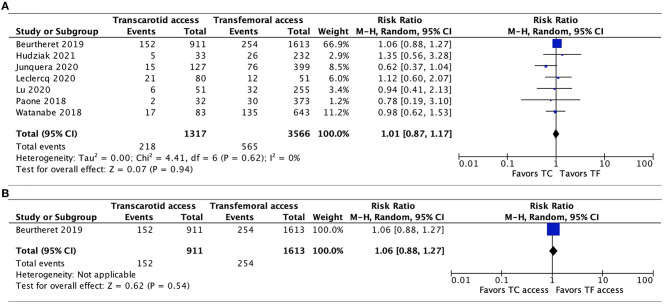
Forest plots comparing permanent pacemaker implantation between TC and TF transcatheter aortic valve replacement procedures. **(A)** Pooled data from all available studies. **(B)** Data from propensity-score matched study. TC, transcarotid; TF, transfemoral.

Seven studies reported the incidence of PPM implantation (11, 20, 22–24, 26, 27). There was no significant difference between TC-TAVR and TF-TAVR, either when all 7 studies were included (RR, 1.01, 95% CI, 0.87–1.17, *I*^2^ = 0%, *p* = 0.94), or in the subgroup of propensity-score matched studies (only data from one study were available, RR, 1.06, 95% CI, 0.88–1.27, *p* = 0.54; Figures 5A,B).

#### Major Bleeding

**Figure 6:**
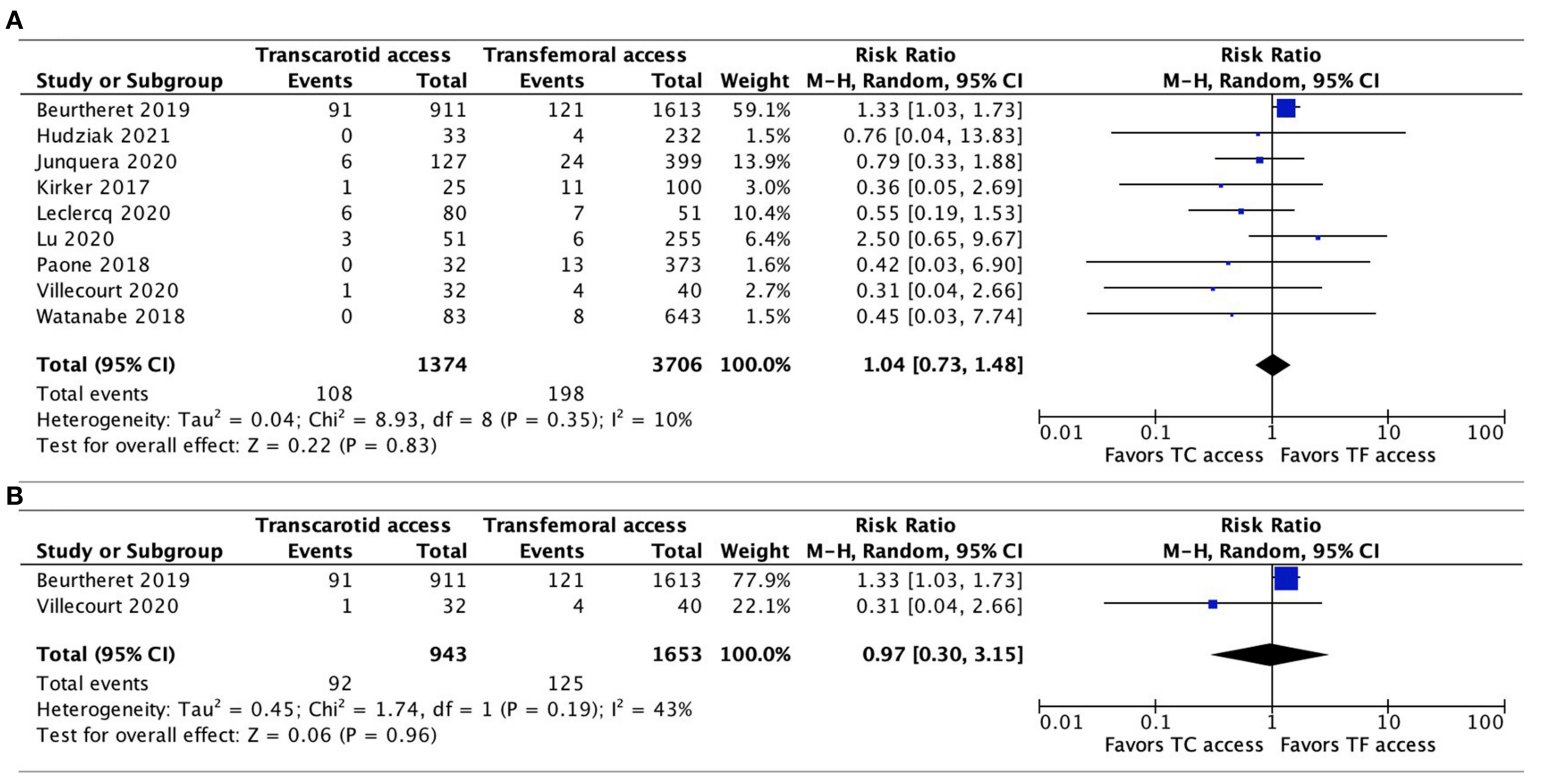
Forest plots comparing major bleeding between TC and TF transcatheter aortic valve replacement procedures. **(A)** Pooled data from all studies. **(B)** Data from propensity-score matched study. TC, transcarotid; TF, transfemoral.

The incidence of major bleeding was reported in all 9 studies, with no significant difference between TC-TAVR and TF-TAVR observed after pooling all the results (RR, 1.04, 95% CI, 0.73–1.48, *I*^2^ = 10%, *p* = 0.83), and pooling only of propensity-score matched studies (RR, 0.97, 95% CI, 0.3–3.15, *I*^2^ = 43%, *p* = 0.96; Figures 6A,B).

#### Acute Kidney Injury

**Figure 7:**
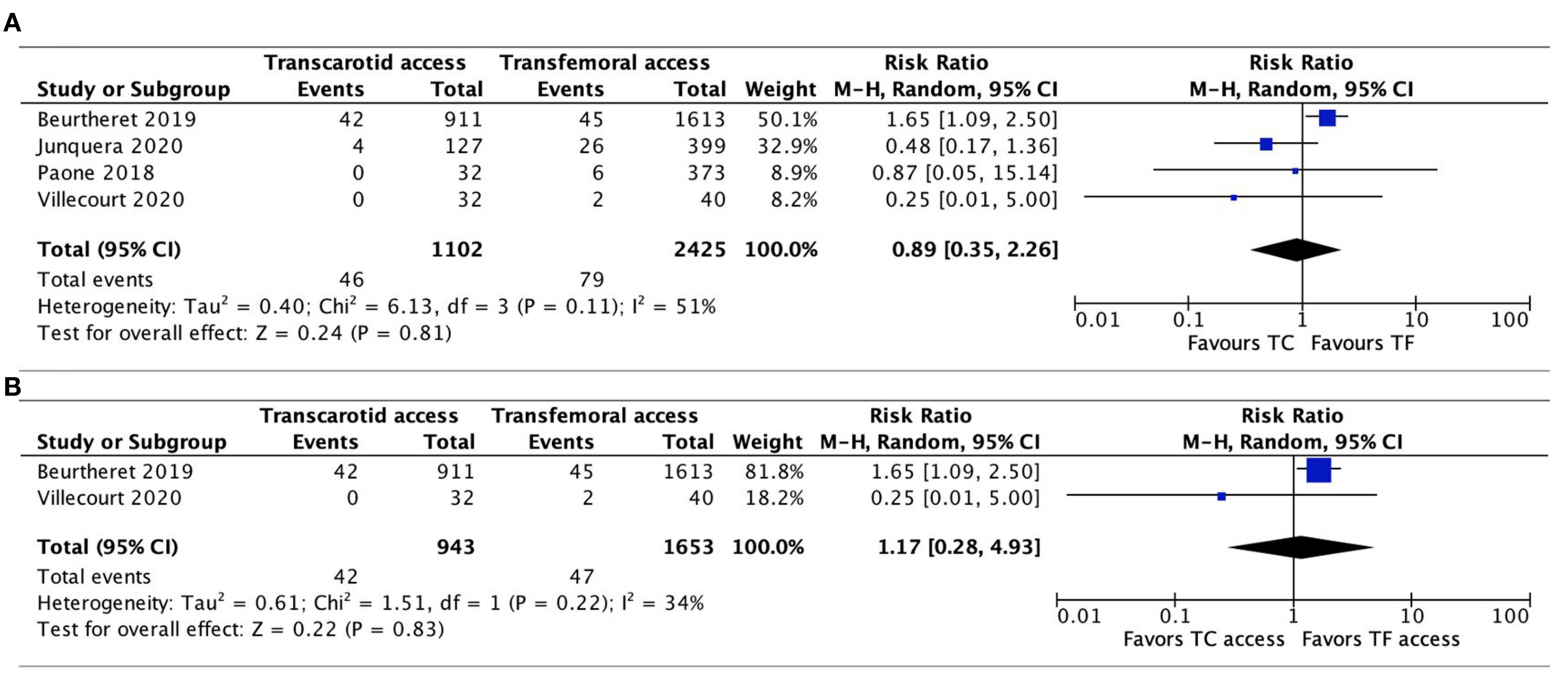
Forest plots comparing acute kidney injury between TC and TF transcatheter aortic valve replacement procedures. **(A)** Pooled data from available studies. **(B)** Data from propensity-score matched studies. TC, transcarotid; TF, transfemoral.

AKI was reported in 4 studies (20, 21, 26, 27), without any significant difference evidenced between TC-TAVR and TF-TAVR in the pooled results (RR, 0.89, 95% CI, 0.35–2.26, *I*^2^ = 51%, *p* = 0.81) and in propensity-score matched studies (RR, 1.17, 95% CI, 0.28–4.93, *I*^2^ = 34%, *p* = 0.83; Figures 7A,B).

## Discussion

This meta-analysis of 1,374 TC patients and 3,706 TF patients is the first to exclusively compare TC-TAVR to TF-TAVR. A previous meta-analysis has pooled data from TC and TSc patients (14), however, some studies have suggested these two pathways yield slightly different outcomes (28, 29).

The main findings of our meta-analysis can be summarized as follows: (1) TC-TAVR was associated with a significantly higher risk of 30-day mortality, (2) TC patients presented a significantly higher risk of neurovascular complications in the propensity-score matched studies, (3) TC-TAVR was associated with a significantly lower risk of major vascular complications, (4) there was no significant difference between the TC and TF accesses regarding the other 30-day outcomes (PPM implantation, major bleeding, or AKI). However, in the propensity-score matched studies, TC-TAVR was no longer associated with a significantly higher risk of 30-day mortality and a lower risk of major vascular complications.

The increased risk of 30-day mortality associated with TC-TAVR is likely related to the higher surgical risk and comorbidity burden TC patients exhibited. Supporting this hypothesis, the association was not found in the subgroup of propensity-score matched studies, where patients' baseline characteristics and surgical risk were similar. Furthermore, the higher surgical risk and cardiovascular disease burden found in TC patients were expected, as these patients, by definition, have more chance to present contraindications to TF-TAVR. These observations regarding the association between increased mortality and higher surgical risk and comorbidity burden in TC-TAVR must be taken with caution: by comparison the same argument was used in some early studies to explain why TAp-TAVR presented with higher mortality, compared with TF-TAVR (30). However, in subanalyses of the randomized PARTNER trial, TAp-TAVR was found to be associated with a significant risk of cardiac mortality, despite the lack of differences regarding baseline population characteristics (31). Another possible limitation to these observations resides in the way surgical risk was assessed in the studies: the Logistic EuroSCORE, EuroSCORE II, and STS score were originally developed for a cardiac surgical setting and, although they are commonly used in TAVR studies, they may have less predictive value in comparing two TAVR interventions.

The reason why the prevalence of PAD in TC-TAVR patients was only 52% and not higher is not clear. Precise definition of PAD was not given in most articles, and it is possible that there was some heterogeneity in the way it was defined and assessed. We have to keep in mind that contraindications to the TF approach, beside PAD, also include, among others: extreme vessel tortuosity or abdominal aortic aneurysms, which would not necessarily be defined as PAD. It is possible that the latter contraindications may account for a significant part of TC-TAVR interventions. Leclercq et al. did report the characteristics of the ilio-femoral vascular access in patients undergoing TC-TAVR: out of 80 patients, 48 (60%) had severe femoral tortuosity or complex aortic access (23). Another possible explanation to the relatively low prevalence of PAD in the TC group is the fact that one single propensity-matched study accounted for nearly two-thirds of patients (911 out of 1,374) undergoing TC-TAVR (20). These 911 patients were matched with corresponding TF-TAVR patients and this may have somewhat biased the patients' baseline characteristics, as prevalence of PAD was relatively low among them (50%). Still, overall, the prevalence of PAD was increased in TC-TAVR patients, when compared with TF-TAVR patients, and this reflected the contraindications to the transfemoral access. Some data suggest the atherosclerotic process may preferentially affect the femoral arteries, more than the carotid arteries (32).

The incidence of 30-day neurovascular complications was significantly higher in TC patients, when considering the propensity-score matched studies, but not when all studies were pooled together (although there was a trend toward significance). This difference may be due to a selection bias resulting from patients' characteristics according to arterial access, when unadjusted studies are included. We also have to consider the heterogeneity concerning the way TC-TAVR interventions were performed, e.g., the side of the CCA that was used, the type of THVs (SE or BE), the surgeon's operative technique, and the modality of anesthesia (general or local): it is unknown if all this may have influenced the risk of stroke. Furthermore, the way neurovascular complications were assessed may be subject to caution: by comparison recent prospective trials regarding TAVR interventions used a very robust evaluation of neurological events [e.g., systematic neurological functional assessment before and after procedure; (33)]. In this regard, the result from the subgroup of propensity-score matched studies is possibly more reliable, as some potential confounding factors due to population differences were controlled. The higher incidence of neurovascular complications observed in this subgroup may be explained by several factors: embolization of CCA plaque due to arterial puncture, access site trauma providing a nidus for thrombosis with subsequent embolization, inadequate collateral perfusion through the circle of Willis and embolization of debris during balloon valvuloplasty or THV implantation (34). These findings are in line with a pilot study, which found TC-TACR to be associated with more abundant and larger subclinical ischemic lesions (assessed by brain magnetic resonance imaging) in the hemisphere of the brain perfused by the CCA that was punctured (35). A thorough evaluation of atherosclerotic plaques before intervention via appropriate imaging exams (e.g., Doppler ultrasound, with exclusion of patients presenting >50% CCA stenosis) and of the functional integrity of the circle of Willis intraoperatively using the CCA clamping test, may contribute to lower the risk of cerebral complications (9, 13). Continuous monitoring of cerebral oximetry throughout the procedure is paramount. By analogy with carotid endarterectomy, some authors propose to abort the intervention if a significant drop of oximetry parameters (>20%) is detected, the limit of 20% being associated with an increased risk of cerebral ischemia (11, 36). Others suggest that local anesthesia with sedation may be preferable over general anesthesia by allowing “real-time” neurological evaluation (37). Although embolic protection systems have been studied in TF-TAVR, to our knowledge, no literature in the setting of TC-TAVR exists (11). Their role in TC patients requires further investigations to determine usefulness in the reduction of the risk of stroke (38).

The risk of major vascular complications was significantly lower in TC patients, in the overall pooled analysis, but not in the propensity-score matched studies analysis. The reason of the difference between the two analyses is not clear, as the risk of vascular complications should in theory only depend on local anatomy and surgical technique. Previous meta-analyses have also found a decreased risk of vascular complications associated with TC-TAVR (39). An explanation may be that, in TC-TAVR, the CCA is approached, cannulated, and reconstructed surgically, while most TF cases are performed percutaneously, which does not allow direct vascular control (1, 40).

Our data showed no significant difference regarding the risk of PPM implantation, major bleeding or AKI. Overall, our results regarding the incidence of 30-day mortality (4.0%), neurovascular complications (3.1%), PPM implantation (16.6%), AKI (4.8%) in the TC group lie in the same range as those of recently published meta-analyses: Bob-Manuel et al. reported respective incidence of 4.2, 5.0, and 15.3% (no data concerning AKI) (12), while Usman et al. described respective incidence of 5.3, 3.4, 15.3, and 3.4% (39). However, our data on major bleeding and vascular complications were different: respectively 7.9 and 1.0% vs. 3.7 and 4.2% for Bob-Manuel et al., and 4.3 and 2.4% for Usman et al. This is mainly explained by the inclusion in our analysis of the study by Beurtheret et al., which had the biggest population sample and reported high rates of major bleeding (10.0%) and low rates of vascular complications (0.2%) (20). The reason for these differences is not clear, but it is worth noting that the bleeding rate was higher in the 2013–2015 period, compared with the 2015–2017 period, suggesting a temporal trend associated with the incidence of that complication. In fact, this “time factor” may have to be taken into account when considering the incidence of all complications associated with TC-TAVR: using a cumulative meta-analysis model, Usman et al. showed that there was a temporal trend of decreasing incidence of stroke/TIA, major vascular complications and AKI for TC-TAVR (39). Possible explanations include the continuing advances in TAVR technology with the development of newer-generation valves with better deliverability, lower profile, an increase in operator expertise, and also to evolving modalities of screening of patients suitable to this approach. Furthermore, whether the difference regarding the types of THV used between TC- and TF-TAVR may have impacted the outcomes is unclear. In a propensity-matched comparison of the two types of THVs in TF-TAVR interventions, no difference was found regarding the risk of stroke, major bleeding, vascular complications, while significant differences existed regarding intra-hospital mortality and PPM (higher incidence with SE-THV) (41).

## Limitations

Our study had some limitations. The most important one is the lack of randomized controlled trials comparing TC and TF-TAVR, hence this meta-analysis included only observational studies; and it is limited by their potential flaws and unidentified sources of bias. Patients' baseline characteristics and surgical risk were not comparable. However, a prospective randomized trial cannot in theory be performed to compare TC- and TF-TAVR as TC patients, by definition, present contraindications to TF-TAVR, and the latter remains the standard approach. Two studies used propensity-score matching, with similar patient demographics in the TC and TF groups, but the other studies had major differences in patient characteristics between the two groups. Furthermore, in two studies, the outcomes were not defined according to the VARC-2 criteria (22, 27). Finally, we included the data of both SE and BE THVs in our analysis. A comparative analysis between these two types of device might reveal one to be superior to the other in TC-TAVR, but this was out of the scope of this study.

## Conclusions

Our study showed that TC-TAVR was associated with an increased risk of 30-day mortality, likely related to a higher surgical risk and higher comorbidity burden, and in the subgroup of propensity-score matched studies, with an increased risk of neurovascular complications. A lower risk of major vascular complications was found in the TC group, and TC-TAVR and TF-TAVR yielded similar results regarding PPM implantation, major bleeding, and AKI. Overall, our results highlight the importance of careful preprocedural patient selection, with a thorough neurovascular evaluation, as well as the need for close periprocedural neurological monitoring. Studies to better define the selection criteria of TC-TAVR are warranted.

## Data Availability Statement

The original contributions presented in the study are included in the article/Supplementary Material, further inquiries can be directed to the corresponding author/s.

## Author Contributions

HL: conceptualization, data curation, formal analysis, methodology, and writing—original draft. PM: methodology and supervision. RH and EE: supervision and validation. SF: data curation, methodology, and supervision. CR, VR, LF, AB, and OM: validation. MK: methodology, supervision, validation, and writing—review and editing. All authors contributed to the article and approved the submitted version.

## Conflict of Interest

SF reports consulting fees from Bayer and Cathworks, EE reports grants from Edwards, VR reports grants from Abbott and Terumo, LF reports research grants from Biotronik, Edwards Lifesciences and Medtronic, OM reports grants from Abbott and Edwards. The remaining authors declare that the research was conducted in the absence of any commercial or financial relationships that could be construed as a potential conflict of interest.

## Supplementary Material

Supplementary Figure 1 Funnel plots for assessment of publication bias in each outcome.

Supplementary Table 1 Search term strategy.

Supplementary Table 2 Quality of assessment using the Newcastle-Ottawa Scale.

Supplementary Table 3 Technical aspects of transcarotid transcatheter aortic valve implantation procedures.

Supplementary Table 4 Type of prosthesis used according to TAVR access.

Supplementary Table 5 Thirty-day complications of patients who underwent TC transcatheter aortic valve replacement.

## References

[1] Lu H Muller O Eeckhout E Monney P Roguelov C Marcucci C. TAVI : une revue de la littérature des voies alternatives à l'accès trans-fémoral. Presse Médicale Form. (2020) 1:249–56. 10.1016/j.lpmfor.2020.04.016

[2] Ye J Cheung A Lichtenstein SV Carere RG Thompson CR Pasupati S. Transapical aortic valve implantation in humans. J Thorac Cardiovasc Surg. (2006) 131:1194–6. 10.1016/j.jtcvs.2006.01.026 16678621

[3] Hayashida K Romano M Lefèvre T Chevalier B Farge A Hovasse T. The transaortic approach for transcatheter aortic valve implantation: a valid alternative to the transapical access in patients with no peripheral vascular option. A single center experience. Eur J Cardiothorac Surg. (2013) 44:692–700. 10.1093/ejcts/ezt037 23439694

[4] Modine T Lemesle G Azzaoui R Sudre A. Aortic valve implantation with the CoreValve ReValving System via left carotid artery access: first case report. J Thorac Cardiovasc Surg. (2010) 140:928–9. 10.1016/j.jtcvs.2010.03.001 20381818

[5] Ruge H Lange R Bleiziffer S Hutter A Mazzitelli D Will A. First successful aortic valve implantation with the CoreValve ReValving System via right subclavian artery access: a case report. Heart Surg Forum. (2008) 11:E323–4. 10.1532/HSF98.20081021 18948247

[6] Pascual I Carro A Avanzas P Hernández-Vaquero D Díaz R Rozado J. Vascular approaches for transcatheter aortic valve implantation. J Thorac Dis. (2017) 9:S478–87. 10.21037/jtd.2017.05.73 28616344 PMC5462718

[7] Lu H Fournier S Namasivayam J Roguelov C Ferrari E Eeckhout E. Transapical approach versus transcervical approach for transcatheter aortic valve replacement: a retrospective monocentric study. Interact Cardio Vasc Thorac Surg. (2020) 31:781–8. 10.1093/icvts/ivaa202 33051655

[8] Allen KB Chhatriwalla AK Cohen D Saxon J Hawa Z Kennedy KF. Transcarotid versus transapical and transaortic access for transcatheter aortic valve replacement. Ann Thorac Surg. (2019) 108:715–22. 10.1016/j.athoracsur.2019.02.007 30880139

[9] Chamandi C Abi-Akar R Rodés-Cabau J Blanchard D Dumont E Spaulding C. Transcarotid compared with other alternative access routes for transcatheter aortic valve replacement. Circ Cardiovasc Interv. (2018) 11:e006388. 10.1161/CIRCINTERVENTIONS.118.006388 30571205

[10] Overtchouk P Folliguet T Pinaud F Fouquet O Pernot M Bonnet G. Transcarotid approach for transcatheter aortic valve replacement with the sapien 3 prosthesis. JACC Cardiovasc Interv. (2019) 12:413–9. 10.1016/j.jcin.2018.11.014 30772290

[11] Lu H Monney P Fournier S Pavon AG Roguelov C Eeckhout E. Transcervical approach versus transfemoral approach for transcatheter aortic valve replacement. Int J Cardiol. (2021) 327:58–62. 10.1016/j.ijcard.2020.11.026 33242507

[12] Bob-Manuel T Almusawi H Rezan T Khaira H Akingbola A Nasir A. Efficacy and safety of transcarotid transcatheter aortic valve replacement: a systematic review. Cardiovasc Revasc Med. (2020) 21:917–26. 10.1016/j.carrev.2019.12.012 31882332

[13] Wee IJY Stonier T Harrison M Choong AMTL. Transcarotid transcatheter aortic valve implantation: a systematic review. J Cardiol. (2018) 71:525–33. 10.1016/j.jjcc.2018.01.010 29499894

[14] Faroux L Junquera L Mohammadi S Del Val D Muntané-Carol G Alperi A. Femoral versus nonfemoral subclavian/carotid arterial access route for transcatheter aortic valve replacement: a systematic review and meta-analysis. J Am Heart Assoc. (2020) 9:e017460. 10.1161/JAHA.120.017460 32990146 PMC7792420

[15] Higgins JPT Thomas J Chandler J Cumpston M Li T Page MJ. Cochrane Handbook for Systematic Reviews of Interventions. 2nd ed. Chichester: John Wiley & Sons (2019). 10.1002/9781119536604

[16] Moher D Liberati A Tetzlaff J Altman DG PRISMA Group. Preferred reporting items for systematic reviews and meta-analyses: the PRISMA statement. Int J Surg. (2010). 8:336–41. 10.1016/j.ijsu.2010.02.007 20171303

[17] Lo CK-L Mertz D Loeb M. Newcastle-Ottawa Scale: comparing reviewers' to authors' assessments. BMC Med Res Methodol. (2014) 14:45. 10.1186/1471-2288-14-45 24690082 PMC4021422

[18] Kappetein AP Head SJ Généreux P Piazza N van Mieghem NM Blackstone EH. Updated standardized endpoint definitions for transcatheter aortic valve implantation: the Valve Academic Research Consortium-2 consensus document. Eur Heart J. (2012) 33:2403–18. 10.1093/eurheartj/ehs255 23026477

[19] Folliguet TA Teiger E Beurtheret S Modine T Lefevre T Van Belle E. Carotid versus femoral access for transcatheter aortic valve implantation: a propensity score inverse probability weighting study. Eur J Cardiothorac Surg. (2019) 56:1140–6. 10.1093/ejcts/ezz216 31365061

[20] Beurtheret S Karam N Resseguier N Houel R Modine T Folliguet T. Femoral versus nonfemoral peripheral access for transcatheter aortic valve replacement. J Am Coll Cardiol. (2019) 74:2728–39. 10.1016/j.jacc.2019.09.054 31779788

[21] Villecourt A Faroux L Muneaux A Tassan-Mangina S Heroguelle V Poncet A. Comparison of clinical outcomes after transcarotid and transsubclavian versus transfemoral transcatheter aortic valve implantation: a propensity-matched analysis. Arch Cardiovasc Dis. (2020) 113:189–98. 10.1016/j.acvd.2020.01.001 32037133

[22] Watanabe M Takahashi S Yamaoka H Sueda T Piperata A Zirphile X. Comparison of transcarotid vs. transfemoral transcatheter aortic valve implantation. Circ J. (2018) 82:2518–22. 10.1253/circj.CJ-18-0530 30068794

[23] Leclercq F Choteau R Cayla G Chamard C Lounes Y Lattuca B. Transcarotid versus transfemoral access in patients undergoing transcatheter aortic valve replacement with complex aortofemoral anatomy. Catheter Cardiovasc Interv. (2020). 10.1002/ccd.29438 33325639

[24] Hudziak D Wojakowski W Malinowski M Gocoł R Zak A Morkisz Ł. Comparison of short-term safety and efficacy of transcarotid and transfemoral access routes for transcatheter aortic valve implantation. Kardiol Pol. (2021) 79:31–8. 10.33963/KP.15697 33293496

[25] Kirker EB Hodson RW Spinelli KJ Korngold EC. The carotid artery as a preferred alternative access route for transcatheter aortic valve replacement. Ann Thorac Surg. (2017) 104:621–9. 10.1016/j.athoracsur.2016.12.030 28274520

[26] Junquera L Kalavrouziotis D Côté M Dumont E Paradis DM DeLarochellière R. Results of transcarotid compared with transfemoral transcatheter aortic valve replacement. J Thorac Cardiovasc Surg. (2020). 10.1016/j.jtcvs.2020.03.091. [Epub ahead of print]. 32387164

[27] Paone G Eng M Kabbani LS Borgi J Peterson E Novitsky B. Transcatheter aortic valve replacement: comparing transfemoral, transcarotid, and transcaval access. Ann Thorac Surg. (2018) 106:1105–12. 10.1016/j.athoracsur.2018.04.029 29758214

[28] Amer MR Mosleh W Joshi S Mather JF El-Mallah W Cheema M. Comparative outcomes of transcarotid and transsubclavian transcatheter aortic valve replacement. Ann Thorac Surg. (2020) 109:49–56. 10.1016/j.athoracsur.2019.05.035 31279787

[29] Amer MR Mosleh W Megaly M Shah T Ooi YS McKay RG. Outcomes of transcarotid versus trans-subclavian transcatheter aortic valve replacement: a systematic review and meta-analysis. Cardiovasc Revasc Med. (2021). 10.1016/j.carrev.2021.01.001. [Epub ahead of print]. 33446436

[30] van der Boon RM Marcheix B Tchetche D Chieffo A Van Mieghem NM Dumonteil N. Transapical versus transfemoral aortic valve implantation: a multicenter collaborative study. Ann Thorac Surg. (2014) 97:22–8. 10.1016/j.athoracsur.2013.09.088 24263012

[31] Elmariah S Fearon WF Inglessis I Vlahakes GJ Lindman BR Alu MC. Transapical transcatheter aortic valve replacement is associated with increased cardiac mortality in patients with left ventricular dysfunction: insights from the PARTNER I Trial. JACC Cardiovasc Interv. (2017) 10:2414–22. 10.1016/j.jcin.2017.09.023 29217004

[32] Laclaustra M Casasnovas JA Fernández-Ortiz A Fuster V León-Latre M Jiménez-Borreguero LJ. Femoral and carotid subclinical atherosclerosis association with risk factors and coronary calcium. J Am Coll Cardiol. (2016) 67:1263–74. 10.1016/j.jacc.2015.12.056 26988945

[33] Popma JJ Deeb GM Yakubov SJ Mumtaz M Gada H O'Hair D. Transcatheter aortic-valve replacement with a self-expanding valve in low-risk patients. N Engl J Med. (2019) 380:1706–15. 10.1056/NEJMoa1816885 30883053

[34] Mylotte D Sudre A Teiger E Obadia JF Lee M Spence M. Transcarotid transcatheter aortic valve replacement: feasibility and safety. JACC Cardiovasc Interv. (2016) 9:472–80. 10.1016/j.jcin.2015.11.045 26965937

[35] Chamandi C Mohammadi S Dumont E Doyle D DeLarochellière R Paradis J-M. Cerebral embolism following transcarotid transcatheter aortic valve replacement. J Am Coll Cardiol. (2018) 71:101–2. 10.1016/j.jacc.2017.10.075 29301616

[36] Ritter JC Green D Slim H Tiwari A Brown J Rashid H. The role of cerebral oximetry in combination with awake testing in patients undergoing carotid endarterectomy under local anaesthesia. Eur J Vasc Endovasc Surg. (2011) 41:599–605. 10.1016/j.ejvs.2010.12.009 21354833

[37] Debry N Delhaye C Azmoun A Ramadan R Fradi S Brenot P. Transcarotid transcatheter aortic valve replacement: general or local anesthesia. JACC Cardiovasc Interv. (2016) 9:2113–20. 10.1016/j.jcin.2016.08.013 27765304

[38] Haussig S Mangner N Dwyer MG Lehmkuhl L Lücke C Woitek F. Effect of a cerebral protection device on brain lesions following transcatheter aortic valve implantation in patients with severe aortic stenosis: the CLEAN-TAVI randomized clinical trial. JAMA. (2016) 316:592–601. 10.1001/jama.2016.10302 27532914

[39] Usman MS Rawasia WF Siddiqi TJ Mujeeb FA Nadeem S Alkhouli M. Meta-analysis evaluating the safety and efficacy of transcarotid transcatheter aortic valve implantation. Am J Cardiol. (2019) 124:1940–6. 10.1016/j.amjcard.2019.09.015 31653356

[40] Overtchouk P Alqdeimat I Coisne A Fattouch K Modine T. Transcarotid approach for TAVI: an optimal alternative to the transfemoral gold standard. Ann Cardiothorac Surg. (2017) 6:555–7. 10.21037/acs.2017.08.04 29062754 PMC5639220

[41] Van Belle E Vincent F Labreuche J Auffret V Debry N Lefèvre T. Balloon-expandable versus self-expanding transcatheter aortic valve replacement: a propensity-matched comparison from the FRANCE-TAVI registry. Circulation. (2020) 141:243–59. 10.1161/CIRCULATIONAHA.120.047270 31736356

