# Supplementary figures and images for: Transcarotid Access Versus Transfemoral Access for Transcatheter Aortic Valve Replacement: A Systematic Review and Meta-Analysis
Source: Front Cardiovasc Med. 2021 May 27;8:687168. doi: 10.3389/fcvm.2021.687168 (PMC8190826; doi:10.3389/fcvm.2021.687168)

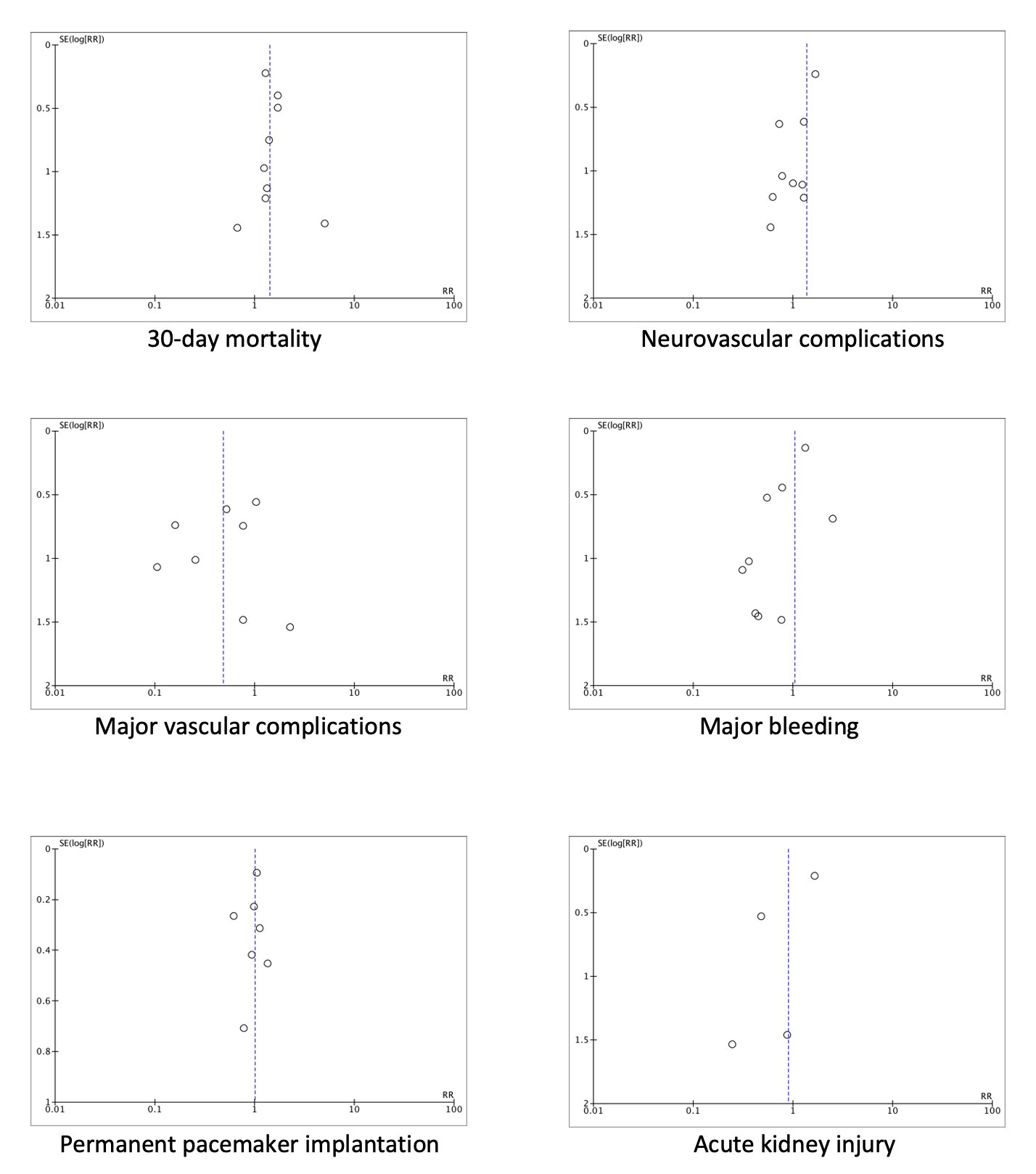

Supplement: Supplementary Figure 1 — Funnel plots for assessment of publication bias in each outcome. [file Image_1.JPEG]
