# Supplementary material for: Transcarotid Access Versus Transfemoral Access for Transcatheter Aortic Valve Replacement: A Systematic Review and Meta-Analysis
Source: Front Cardiovasc Med. 2021 May 27;8:687168. doi: 10.3389/fcvm.2021.687168 (PMC8190826; doi:10.3389/fcvm.2021.687168)
Supplement: Supplementary Table 1 — Search term strategy. [file Data_Sheet_1.docx]

**Supplementary Table 1**. Search term strategy.

| PubMed/MEDLINE | (Transcatheter Aortic Valve Replacement OR Percutaneous aortic OR Transcatheter aortic valve implantation OR TAVI OR TAVR) AND (transcarotid OR transcervical OR trans-carotid OR carotid) |
| --- | --- |
| EMBASE | ('transcatheter aortic valve implantation'/exp OR 'transcatheter aortic valve implantation') AND ('transcarotid' OR 'transcervical’ OR 'trans-carotid' OR 'carotid’) |

**Supplementary Table 2**. Quality of assessment using the Newcastle-Ottawa Scale.

| Study | Selection | | | Comparability | Outcomes | | | Total score |
| --- | --- | --- | --- | --- | --- | --- | --- | --- |
|  | Representativeness of exposed cohort | Selection of non-exposed cohort | Ascertainment of exposure | Comparability of cohorts on the basis of the design or analysis | Assessment of outcomes | Length of follow-up of cohorts | Adequacy of follow-up of cohorts |  |
| Kirker 2017 [25] | * | * | * | * | * | * | * | *******  (7) |
| Paone 2018 [27] | * | * | * | * | - | * | * | ******  (6) |
| Watanabe 2018 [22] | * | * | * | * | - | * | * | ******  (6) |
| Beurtheret 2019 [20] | * | * | * | ** | * | * | * | *******  (7) |
| Villecourt 2020 [21] | * | * | * | ** | * | * | * | ********  (8) |
| Junquera 2020 [26] | * | * | * | * | * | * | * | *******  (7) |
| Lu 2020 [11] | * | * | * | * | * | * | * | *******  (7) |
| Leclercq 2020 [23] | * | * | * | * | * | * | * | *******  (7) |
| Hudziak 2021 [24] | * | * | * | * | * | * | * | *******  (7) |

The lack of star for the assessment of outcomes for Paone et al. and Watanabe et al. is justified by the fact that the VARC-2 criteria were not used.

**Supplementary Table 3**. Technical aspects of transcarotid transcatheter aortic valve implantation procedures.

| **Authors/year** | **Left carotid access (%)** | **Valve type (%)** | | **GA (%)** | **Intra-operative neuro-monitoring** | **Intervention duration (min)** |
| --- | --- | --- | --- | --- | --- | --- |
|  |  | **SE prosthesis** | **BE prosthesis** |  |  |  |
| Kirker 2017 [25] | 15.0 | 84.0 | 16.0 | 100 | - CCA cross clamping  test  - Continuous cerebral oximetry | 75 (16-19) |
| Paone 2018 [27] | 21.8 | 93.8 | 6.2 | 100 | No cerebral monitoring | Unknown |
| Watanabe 2018  [22] | 100 | 54.2 | 45.8 | 100 | - CCA cross clamping  test  - Continuous cerebral oximetry | 77±29 |
| Beurtheret 2019 [20] | Unknown | 46.3 | 53.7 | Unknown | Unknown | Unknown |
| Villecourt 2020  [21] | Unknown | 84.4 | 15.6 | Unknown | - CCA cross clamping  test  - Continuous cerebral oximetry | Unknown |
| Junquera 2020  [26] | 92.0 | 61.4 | 38.6 | 99.2 | Continuous cerebral saturation assessment  with the INVOS system (Medtronic, Minneapolis, Minn) | 65 (55-76) |
| Lu 2020 [11] | 2.0 | 0.0 | 100.0 | 96.1 | - CCA cross clamping  test  - Continuous cerebral oximetry | 74 (53-99) |
| Leclercq 2020 [23] | 76.0 | 82.3 | 18.7 | 98.7 | Unknown | 71±25 |
| Hudziak 2021 [24] | 97.0 | 24.2 | 75.8 | 100 | Continuous cerebral  oximetry (INVOS 5100C, Medtronic, Dublin,  Ireland) | 65 (60–80) |
| Total | 70.8 | 46.3 | 53.7 | 99.1 |  |  |

BE : balloon-expandable, SE : self-expendable, GA : general anesthesia, CCA : common carotid artery

**Supplementary Table 4**. Type of prosthesis used according to TAVR access.

|  | **TC-TAVR** | **TF-TAVR** | **P value** |
| --- | --- | --- | --- |
| SE prosthesis (%) | 625 (46.3%) | 1297 (36.0%) | <0.001 |
| BE prosthesis (%) | 725 (53.7%) | 2309 (64.0%) |  |

BE : balloon-expandable, SE : self-expendable, TC : transcarotid, TAVR : transcatheter aortic valve replacement, TF transfemoral.

[25] was not included in the analysis, because of missing data for TF-TAVR patients.

**Supplementary Table 5**. 30-day complications of patients who underwent TC transcatheter aortic valve replacement.

| **Authors/year** | **TIA or stroke (%)** | **Major bleeding (%)** | **Major vascular complication (%)** | **Acute kidney injury (%)** | **PM implantation (%)** | **Length of stay (days)** | **30-day mortality (%)** |
| --- | --- | --- | --- | --- | --- | --- | --- |
| Kirker 2017 [25] | 4.0 | 4.0 | 0.0 | Unknown | Unknown | 3.0 (2.0-3.0) | 4.0 |
| Paone 2018 [27] | 0.0 | 0.0 | 0.0 | 0.0 | 6.3 | 3.0 (2.0-20.0) | 0.0 |
| Watanabe 2018 [22] | 2.4 | 0.0 | 1.2 | Unknown | 20.5 | 12.4±7.7 | 8.4 |
| Beurtheret 2019 [20] | 3.6 | 10.0 | 0.2 | 5.4 | 16.7 | Unknown | 3.7 |
| Villecourt 2020 [21] | 3.1 | 3.1 | 15.6 | 0 | Unknown | Unknown | 6.2 |
| Junquera 2020 [26] | 2.4 | 4.7 | 2.4 | 3.2 | 11.8 | 5.7±3.4 | 4.8 |
| Lu 2020 [11] | 2.0 | 5.9 | 3.9 | Unknown | 11.8 | 6.0 (4.0-9.0) | 2.0 |
| Leclercq 2020 [23] | 2.5 | 7.5 | 1.3 | Unknown | 26.2 | 6.8±4.3 | 2.6 |
| Hudziak 2021 [24] | 0.0 | 0.0 | 0.0 | Unknown | 15.1 | 6.0 (6.0-7.0) | 6.1 |
| Total | 3.1 | 7.9 | 1.0 | 4.8 | 16.6 |  | 4.0 |

Lengths of stay are expressed as mean ± SD or median (IQR). TC: transcarotid, TIA: transient ischemic attack, PM: pacemaker.
